# Gender differences in perceptions of leadership and their influence on motivation among faculty members of Taif University

**DOI:** 10.3389/fpsyg.2024.1476526

**Published:** 2024-11-14

**Authors:** Fawziah B. Alharthi

**Affiliations:** Department of Management, College of Business Administration, Taif University, Taif, Saudi Arabia

**Keywords:** gender differences, leadership perceptions, faculty motivation, Islamic higher education, transformational leadership, transactional leadership

## Abstract

This study investigates gender differences in perceptions of leadership styles and their impact on faculty motivation at Taif University, Saudi Arabia, within the context of Islamic higher education. Utilizing a quantitative research method approach, the researcher gathered survey responses from 74 faculty members at Taif University. The investigation focused on three main areas: the significant gender differences in perceptions of leadership styles among faculty members at Taif University; the tendency of female faculty members to perceive transformational leadership styles more positively than their male counterparts; and the more favorable response of male faculty members to transactional leadership styles compared to female faculty members. The results of this study revealed significant gender differences, with female faculty members showing a preference for transformational leadership, while male faculty members responded more positively to transactional leadership. By adopting these findings, leaders at Taif University can participate positively on faculty members' motivation. In addition to that leadership practices can move toward sustainability, ensuring the continuous development of faculty, students, and resources. These findings contribute to a deeper understanding of gender-based perceptions of leadership in an Islamic higher education setting and offer strategies for enhancing faculty motivation through tailored leadership approaches.

## 1 Introduction

Leadership is a crucial component in organizational psychology, significantly influencing employee motivation and organizational development (Al Mustofa, 2022). It is often viewed as a strategy for motivating and directing individuals toward specific goals, making it essential for achieving organizational objectives (Kouzes and Posner, 2016). This study focuses on transformational and transactional leadership styles, widely examined in leadership research (Bass, 1985; Avolio and Bass, 1991; Amirul and Daud, 2012), within the context of Islamic higher education (Alharthi, 2023; Saad Alessa, 2021).

Over several decades many research studies have been conducted based on Burns (1978) transformational and transactional leadership theory. Transformational leadership inspires followers by creating a vision, fostering intellectual stimulation, and addressing individual needs, leading to higher motivation and performance (Burns, 1978; Bass and Riggio, 2006). Transactional leadership operates on a system of rewards and penalties, effective for short-term goals but not fostering long-term engagement (Burns, 1978; Judge and Piccolo, 2004).

In Islamic higher education, leadership practices are shaped by principles of trust, ethical behavior, and respect, which positively influence motivation by creating a supportive work environment (Ali, 2009; Beekun and Badawi, 1999). These principles include justice, consultation (shura), and accountability (Ahmad and Fontaine, 2011; Rafiki, 2020).

Saudi Arabia's Vision 2030 includes education as one of its primary objectives. The nation hopes to place at least five of its universities in the top 200 globally, boost parental involvement in the educational process, align the educational system with the needs of the market, and make sure that the system promotes the use of built-in economic opportunities (Vision 2030, 2016). Women in particular have been given more focus in education sector in unison with the fourth and fifth goals of the United Nations Sustainable Development (UNSDs).

Given that higher education is the subject of this study, a number of studies have been conducted in the literature that frequently encourage universities to become more involved in sustainabile education (Leal Filho et al., 2020; Abdelwahed et al., 2022; Bakr, 2022). Universities also need to set an example for the rest of society by being committed and responsible organizational role models.

Leadership attributes are viewed differently by men and women (Eagly and Johannesen-Schmidt, 2001). While men prefer transactional leadership, women may react better to transformational leadership (Eagly and Carli, 2003; Eagly and Johannesen-Schmidt, 2001). Developing successful leadership strategies for a variety of faculty demands requires an understanding of these preferences.

Leadership can contribute to faculty members' motivation. Motivation in academia, both intrinsic and extrinsic, is critical for faculty performance and job satisfaction. Effective leadership enhances motivation, contributing to higher productivity and engagement (Deci and Ryan, 2000; Herzberg, 1968; Vroom, 1964). Transformational leadership has been shown to have significant positive outcomes on employee motivation in various settings (Judge and Piccolo, 2004; Yukl, 2013).

Within the framework of Islamic higher education, this study examines how faculty motivation at Taif University in Saudi Arabia is affected by gender differences in perceptions of leadership styles. The results of this study contribute to the body of literature regarding both leadership styles and gender, and how faculty members perceive each in the workplace.

## 2 Research gap

There is limited understanding of how gender-based differences in leadership perceptions impact faculty motivation within Islamic higher education, particularly at Taif University. While much research focuses on Western contexts, few studies explore how Islamic principles and cultural factors intersect with gender to shape leadership and motivation in Saudi Arabian universities. Studies such as Alsubaie and Jones (2017) and Al-Asfour et al. (2017) indicated the need for further research on gender dynamics in Saudi higher education.

Al-Saggaf and Simmons (2020) highlighted the role of cultural and religious norms in shaping leadership styles and their reception among faculty. Al-Harthi (2021) pointed out the specific challenges faced by female faculty in attaining leadership positions, affecting their motivation and job satisfaction.

This study aims to fill this gap by examining these gender-based differences in perceptions of leadership styles and their effects on faculty motivation at Taif University.

### 2.1 Research question

Gender is an important variable that must be examined with regard to optimizing leadership effectiveness. This investigative study aims to outline influences held by gender in leadership perceptions and its impact on faculty motivation at Taif University. The following research question will be considered and expanded.

How do gender differences in perceptions of leadership styles influence faculty motivation at Taif University, within the context of Islamic higher education?

### 2.2 Objectives of the research

This study aims to:

- Examine the gender-based differences in how faculty members at Taif University perceive various leadership styles.
- Investigate whether female faculty members at Taif University have a more favorable view of transformational leadership styles compared to their male counterparts.
- Assess whether male faculty members at Taif University show a stronger preference for transactional leadership styles compared to female faculty members.

## 3 Research design and sampling

This study employed a quantitative research method, utilizing survey data to address the research questions. The survey captured faculty perceptions of leadership styles and motivation. A quantitative design was used by the researcher in order to increase the number of participants in the study as qualitative research study's conclusions are frequently inductive rather than deductive. The study was conducted at Taif University's main campus, targeting teaching faculty members across 13 colleges. The study involved 74 faculty member responses. Participants were voluntary.

## 4 Data collection and analysis

Data collection involved a survey questionnaire capturing faculty perceptions of leadership styles and motivation. The main instrument used in this research study consisted of closed-ended questionnaires and had three sections. Questions concerning faculty members' perceptions of leadership styles and practices were asked in the first section. The motivation of the faculty members was covered in the second section. The demographic variables covered in the last section were age, gender, education level, and previous experience at Taif University. The Multifactor Leadership Questionnaire (MLQ) developed by Avolio and Bass (1995) and the Work Extrinsic and Intrinsic Motivation Scale (WEIMS) established by Tremblay et al. (2009) were employed in this study.

The data were analyzed using SPSS, employing various statistical tools such as descriptive statistics, frequencies, comparisons, correlations, and multiple regression analyses.

### 4.1 Age and gender distribution of faculty members

Table 1 presents the age and gender distribution of faculty members at Taif University. The data indicates that the majority of participants fall within the 30–39 age range, with the 35–39 age group having the highest representation, comprising 33 individuals. Among these, 20 are female, and 13 are male.

**Table 1:** Age and gender cross-tabulation.

**Age group**	**Female**	**Male**	**Total**
25–29	6	1	7
30–34	15	11	26
35–39	20	13	33
40 or more	5	3	8
Total	46	28	74

In the 30–34 age group, there are 26 faculty members, with females (15) outnumbering males (11). The youngest age group, 25–29, consists of 7 individuals, with a significant female majority of 6 compared to just 1 male. The oldest age group, 40 or more, has 8 participants, with 5 females and 3 males.

Overall, the data reveals a higher number of female faculty members (46) compared to male faculty members (28). This trend is consistent across all age groups, with females consistently outnumbering males. This distribution suggests that gender differences in perceptions of leadership styles and their impact on motivation may be influenced by the predominance of female faculty members, particularly in the middle age ranges where the bulk of the participants fall.

**Figure 1:**
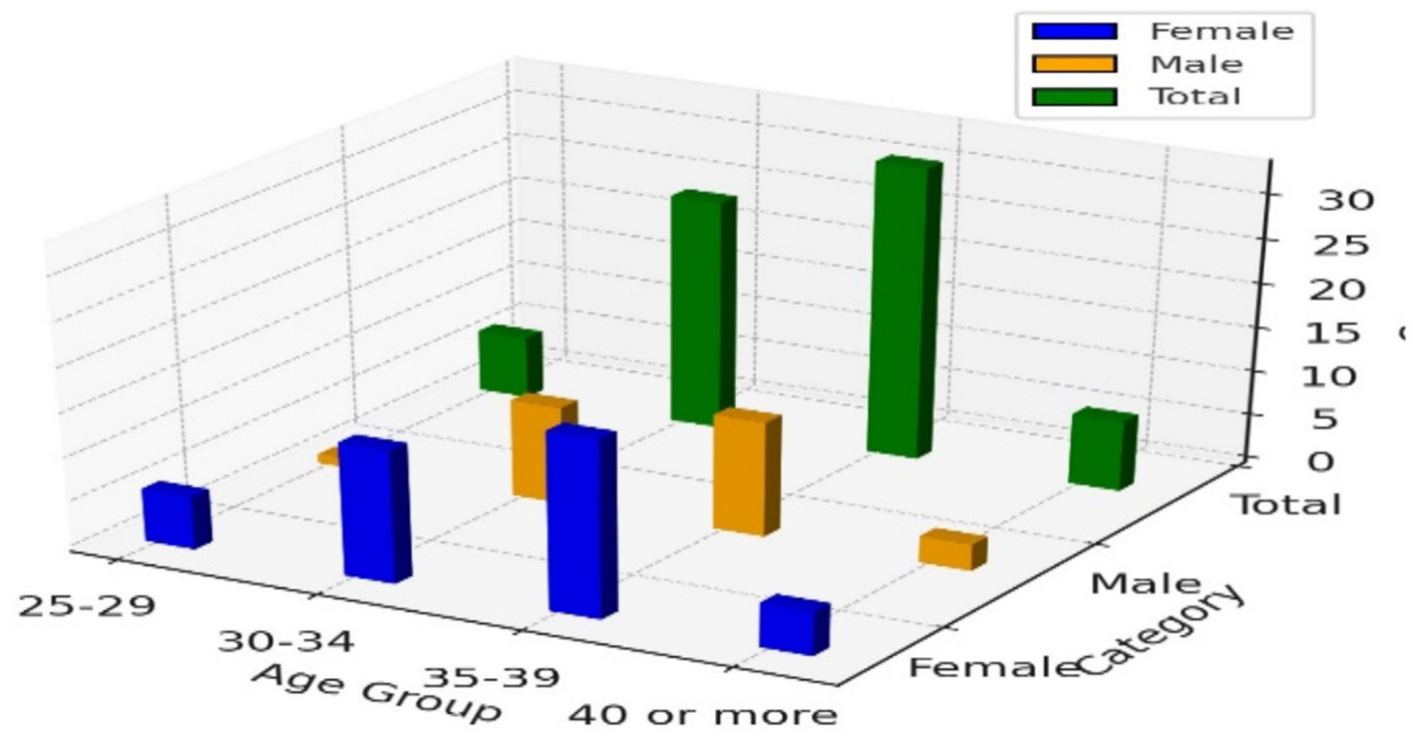
3D bar chart for age and gender.

Figure 1 shows a higher number of female faculty members compared to males across all age groups at Taif University.

### 4.2 Gender and qualifications

Table 2 presents the gender and qualifications of the sample. Among female faculty members, 54.3% have a PhD., 37.0% have a Master's degree, and 8.7% hold a Bachelor's degree. For male faculty members, 50.0% have a PhD., 46.4% have a Master's degree, and 3.6% hold a Bachelor's degree. This indicates that female faculty members are more likely to have a PhD. compared to their male counterparts, while a higher proportion of male faculty have Master's degrees.

**Table 2:** Gender and qualifications cross-tabulation.

**Gender**	**Bachelor**	**Master**	**PhD**	**Total**
Female	4	17	25	46
Male	1	13	14	28
Total	5	30	39	74

**Figure 2:**
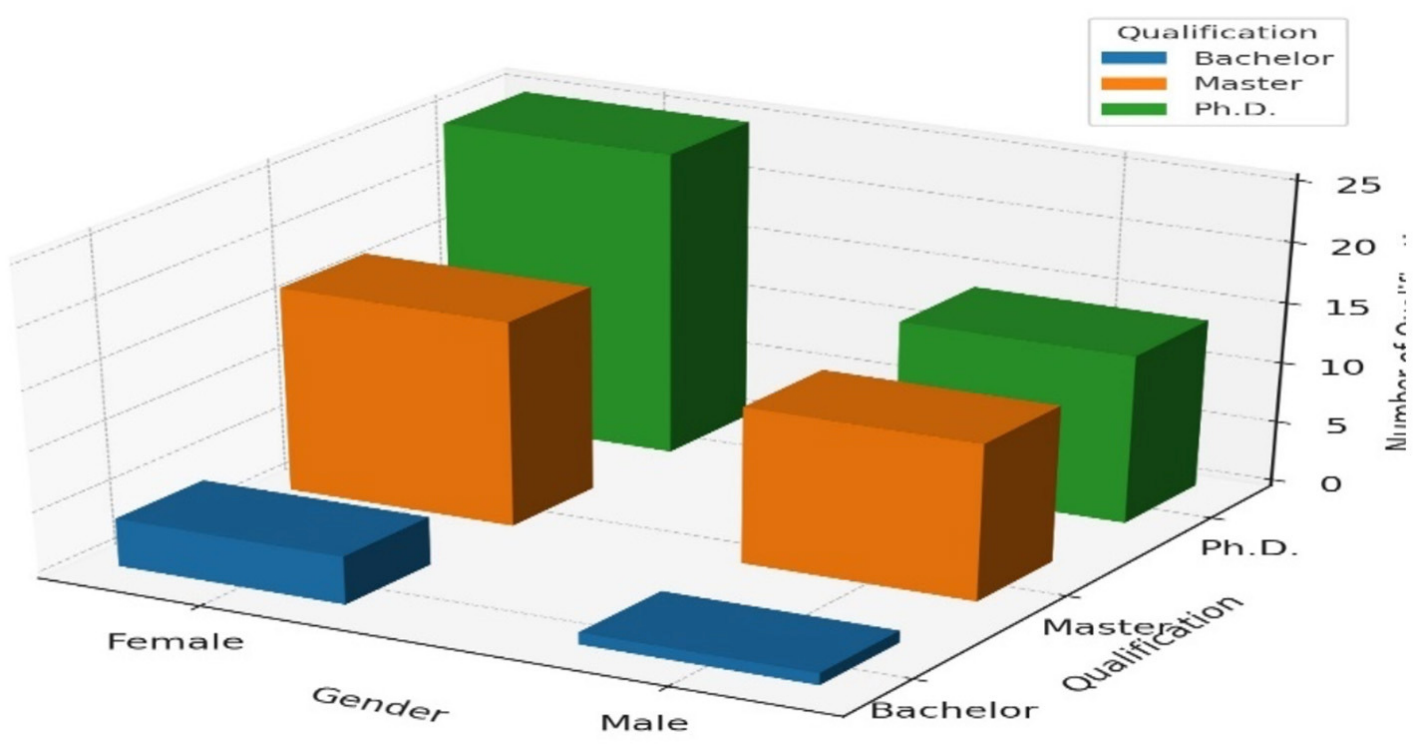
3D bar chart for gender and qualifications.

Figure 2 shows a 3D bar chart cross-tabulating gender (female, male) and education level (Bachelor, Master, Ph.D.), illustrating the count of individuals in each category.

### 4.3 Tenure of faculty members

Table 3 illustrates the relationship between the education levels (Bachelor, Master, Ph.D.) and the years of service among faculty members at Taif University.

- 1–5 Years of Service: Out of 23 faculty members, the majority (13) hold a Master's degree, followed by 8 with Ph.D.s and 2 with Bachelor's degrees. This suggests that relatively new faculty members predominantly possess Master's degrees.
- 6–10 Years of Service: Among the 23 faculty members in this category, 12 hold Ph.D.s, 9 hold Master's degrees, and 2 hold Bachelor's degrees. This shows a trend toward higher qualifications as the years of service increase, with a notable number holding Ph.D.s.
- Less than 1 Year of Service: Of the 3 faculty members in this group, 2 hold Master's degrees, and 1 holds a Bachelor's degree. This indicates that the newest faculty members are mostly Master's degree holders, with no Ph.D. holders in this early service stage.
- More than 10 Years of Service: Among the 25 faculty members, 19 hold Ph.D.s, 6 hold Master's degrees, and none hold Bachelor's degrees. This highlights a significant trend where long-serving faculty members are highly qualified, predominantly with Ph.D. degrees.

**Table 3:** Education level and years of service cross-tabulation.

**Service educational institution**	**Qualification**			
	**Bachelor**	**Master**	**Ph.D**.	**Total**
1–5 years	2	13	8	23
6–10 years	2	9	12	23
<1 year	1	2	0	3
More than 10 years	0	6	19	25
Total	5	30	39	74

**Figure 3:**
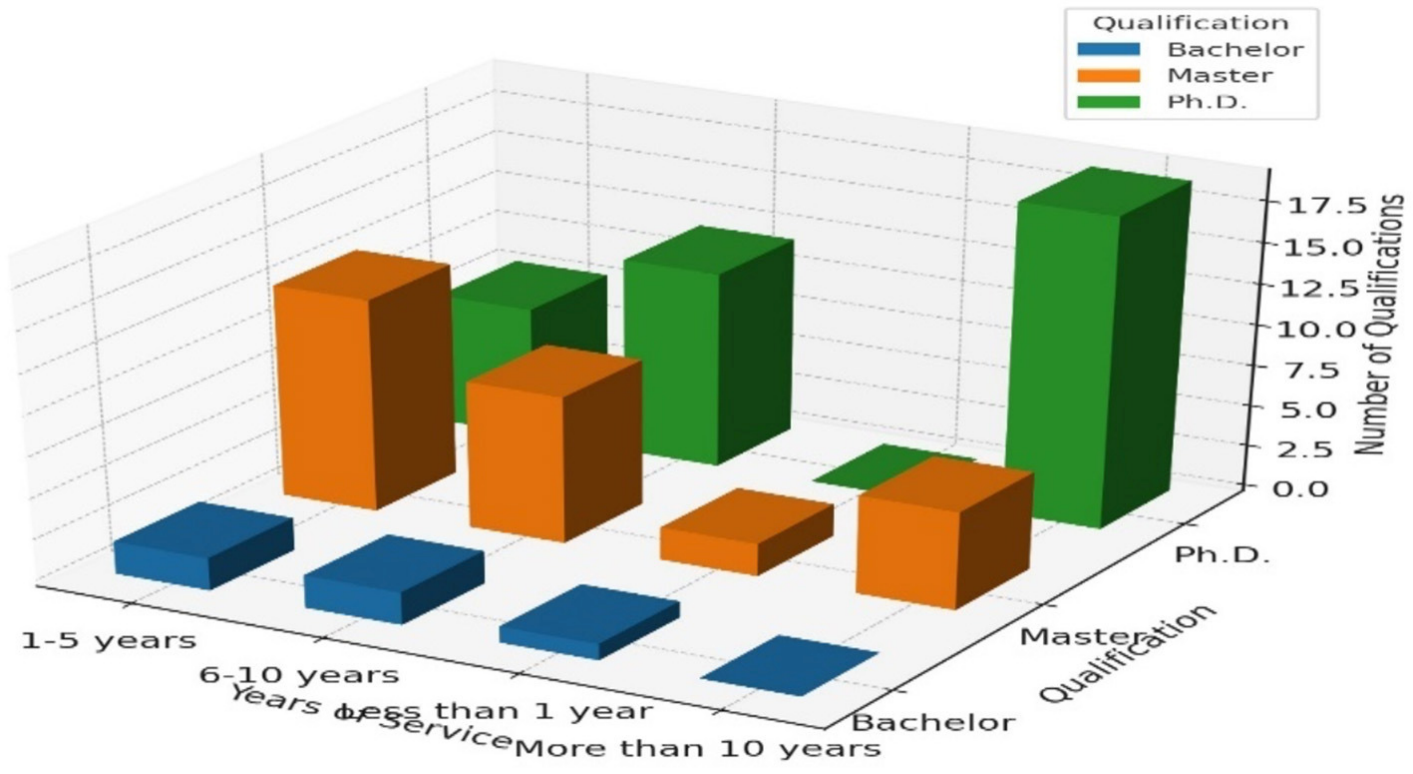
3D bar chart for education level and years of service.

The cross-tabulation above shows that longer service durations correlate with higher academic qualifications, particularly Ph.D.s, highlighting the importance of continuous academic advancement at Taif University. Figure 3 shows the 3D graph representing the cross-tabulation of education level and years of service among faculty members of university.

## 5 Research hypotheses on gender-based leadership perceptions

The purpose of this study is to explore the differences in leadership style perceptions based on gender among faculty members at Taif University. To achieve this, the following research hypotheses have been formulated:

**Hypothesis 1:** *H*_1_**:** There is significant gender-based differences in the perception of transformational leadership among faculty members at Taif University. This hypothesis aims to investigate whether male and female faculty members perceive transformational leadership differently. Transformational leadership involves inspiring and motivating employees to exceed expectations by focusing on long-term goals and personal growth. Understanding if gender influences these perceptions can help tailor leadership development programs more effectively.
**Hypothesis 2:** *H*_2_**:** Female faculty members have a significantly higher preference for transformational leadership styles compared to male faculty members. Building on *H*_1_, this hypothesis specifically predicts that if female faculty members will show a stronger preference for transformational leadership. This means that female faculty members are expected to value leadership behaviors that are visionary, empowering, and supportive of personal and professional development more than their male counterparts.
**Hypothesis 3:** *H*_3_**:** Male faculty members have a significantly higher preference for transactional leadership styles compared to female faculty members. This hypothesis aims to investigate whether male faculty members prefer transactional leadership more than female faculty members. Transactional leadership focuses on routine, supervision, and performance-related rewards and penalties. By examining this hypothesis, the study aims to identify if men are more inclined toward a leadership style that emphasizes structured tasks, clear expectations, and direct rewards for meeting specific goals.

### 5.1 Statistical analysis of leadership style perceptions

Table 4 indicates that there is a significant gender-based difference in the perception of transformational leadership among faculty members at Taif University. Female faculty members have a higher mean score (M = 4.3, SD = 0.6) compared to their male counterparts (M = 3.9, SD = 0.7). The independent samples *t-*test results (t = 3.56, *p* < 0.001) support the hypothesis *H*_1_ that there is significant gender-based differences in the perception of transformational leadership. This result also confirms hypothesis *H*_2_, indicating that female faculty members have a significantly higher preference for transformational leadership styles compared to male faculty members.

**Table 4:** Perceptions of leadership styles by gender among faculty members.

**Leadership style**	**Gender**	**n**	**Mean**	**Standard deviation (SD)**	***t*-value**	***p*-value**
Transformational	Female	46	4.3	0.6	3.56	<0.001
	Male	28	3.9	0.7		
Transactional	Female	46	3.7	0.8	−2.45	0.015
	Male	28	4.1	0.9		

For transactional leadership, the mean score for male faculty members is higher (M = 4.1, SD = 0.9) than that of female faculty members (Mean = 3.7, SD = 0.8). The *t*-test Statistic results (t = −2.45, *p* = 0.015) show a significant difference, suggesting that male faculty members have a higher preference for transactional leadership styles. This finding supports hypothesis *H*_3_.

### 5.2 ANOVA results for perceptions of transformational leadership by gender

The ANOVA results presented in Table 5 provide further insights into the gender-based differences in perceptions of transformational leadership among faculty members at Taif University. The analysis shows that there is a statistically significant difference between the genders. The sum of squares between groups is 8.64 with 1 degree of freedom, resulting in a mean square of 8.64. The F-value is 5.78 with a *p-*value of 0.019. This indicates a significant difference in transformational leadership perceptions between female and male faculty members. The sum of squares within groups is 108.48 with 72 degrees of freedom, yielding a mean square of 1.51. The total sum of squares is 117.12 with 73 degrees of freedom.

**Table 5:** ANOVA results for perceptions of transformational leadership by gender.

**Source**	**Sum of squares**	**Df**	**Mean square**	**F-value**	***p*-value**
Between groups	8.64	1	8.64	5.78	0.019
Within groups	108.48	72	1.51		
Total	117.12	73			

The significant F-value (5.78) and *p*-value (0.019) suggest that gender has a meaningful impact on how transformational leadership is perceived among faculty members at Taif University. This supports the hypothesis that there is significant gender-based differences in the perception of transformational leadership (*H*_1_).

### 5.3 Chi-square test for leadership style preferences by gender

The chi-square test results in Table 6 indicate significant differences in leadership style preferences between genders.

**Table 6:** Chi-square test for preference of leadership style by gender.

**Leadership style**	**Gender**	**Observed (O)**	**Expected (E)**	**(*O*−*E*)^2^/*E***	**Chi-square (χ2)**
Transformational	Female	30	37	1.32	
	Male	18	11	4.45	
Transactional	Female	16	9	5.44	11.21
	Male	10	17	2.89	

For transformational leadership style, female faculty members had observed counts of 30 vs. an expected 37, resulting in a chi-square value of 1.32. Male faculty members had observed counts of 18 vs. an expected 11, with a chi-square value of 4.45.

For transactional leadership style, female faculty members had observed counts of 16 vs. an expected 9, resulting in a chi-square value of 5.44. Male faculty members had observed counts of 10 vs. an expected 17, with a chi-square value of 2.89.

The total chi-square value for transactional leadership is 11.21, suggesting a significant gender difference in leadership style preferences, with female faculty members showing a stronger preference for transformational leadership and male faculty members for transactional leadership.

### 5.4 Reliability analysis

Table 7 presents the results of the reliability analysis for the transformational and transactional leadership styles among female and male faculty members at Taif University. Cronbach's Alpha was used to assess the internal consistency of the survey items for each leadership style by gender.

**Table 7:** Reliability analysis (Cronbach's alpha).

**Leadership style**	**Gender**	**Number of items**	**Sample size (n)**	**Cronbach's alpha**
Transformational	Female	20	46	0.87
	Male	20	28	0.85
Transactional	Female	8	46	0.83
	Male	8	28	0.80

#### 5.4.1 Transformational leadership

**Female:** The Cronbach's Alpha for transformational leadership among female faculty members is 0.87, indicating high internal consistency and reliability of the items measuring this leadership style.

**Male:** The Cronbach's Alpha for transformational leadership among male faculty members is 0.85, also indicating high reliability.

The high reliability scores for both female and male faculty members support the hypothesis (*H*_1_) that perceptions of transformational leadership can be consistently measured across genders. The slight difference in alpha values further supports hypothesis (*H*_2_), suggesting that female faculty members might have a more cohesive perception of transformational leadership.

#### 5.4.2 Transactional leadership

**Female:** The Cronbach's Alpha for transactional leadership among female faculty members is 0.83, indicating good internal consistency.

**Male:** The Cronbach's Alpha for transactional leadership among male faculty members is 0.80, indicating acceptable reliability.

The reliability scores for transactional leadership among both genders support the hypothesis (*H*_1_) as well, affirming the consistency in measuring perceptions of this leadership style. The slightly lower alpha value for males compared to females may indicate a more varied perception among male faculty members, aligning with hypothesis (*H*_3_) that male faculty members show a higher preference for transactional leadership styles.

Overall, the reliability analysis demonstrates that the survey items used to measure both transformational and transactional leadership styles are reliable and consistent across genders, providing robust support for the study's hypotheses.

### 5.5 Statistical analysis of path analysis for direct paths

Table 8 presents the results of the path analysis, which examines the direct relationships between gender and leadership style preferences among faculty members at Taif University.

**Table 8:** Path analysis for direct paths.

**Pathway**	**Coefficient (β)**	***p*-value**
Female → Transformational Leadership	0.35	<0.001
Male → Transactional Leadership	−0.28	0.008

The coefficient of 0.35 indicates a positive and significant direct relationship between gender and transformational leadership, with female faculty members showing a higher preference for transformational leadership styles. The *p*-value of <0.001 suggests that this relationship is statistically significant, providing strong support for hypothesis (*H*_2_). This finding confirms that female faculty members are more likely to favor transformational leadership compared to their male counterparts.

The coefficient of −0.28 indicates a negative and significant direct relationship between gender and transactional leadership, with male faculty members showing a higher preference for transactional leadership styles. The *p-*value of 0.008 indicates that this relationship is statistically significant, supporting hypothesis (*H*_3_). This result suggests that male faculty members are more inclined toward transactional leadership compared to female faculty members.

### 5.6 Multiple regression model

To further explore the influence of gender on leadership style preferences among faculty members at Taif University, we formulated and tested multiple regression models with transformational and transactional leadership as the dependent variables (Y) and gender (G) as the primary independent variable, along with control variables such as age (A), years of experience (E), and academic rank (R).

Model 1: Predicting Transformational Leadership


$$Y_{Transform}=\beta_{0}+\beta_{1}\left(G\right)+\beta_{2}\left(A\right)+\beta_{3}\left(E\right)+\beta_{4}\left(R\right)+\varepsilon_{i}\;$$

From Table 9, the coefficient for gender (0.42) is significant (*p* < 0.001), indicating that being female is associated with a higher preference for transformational leadership compared to males, holding other factors constant. This supports hypothesis *H*_2_.

**Table 9:** Regression results for transformational leadership.

**Variable**	**β**	**Standard error (SE)**	**t-value**	***p*-value**
Intercept	2.85	0.35	8.14	<0.001
Gender	0.42	0.10	4.20	<0.001
Age	0.05	0.03	1.67	0.098
Years of experience	0.03	0.02	1.50	0.137
Academic rank	0.08	0.05	1.60	0.112

Model 2: Predicting Transactional Leadership


$$Y_{Transact}=\beta_{0}+\beta_{1}\left(G\right)+\beta_{2}\left(A\right)+\beta_{3}\left(E\right)+\beta_{4}\left(R\right)+\varepsilon_{i}\;$$

From Table 10, the coefficient for gender (-0.35) is significant (*p* = 0.005), indicating that being male is associated with a higher preference for transactional leadership compared to females, holding other factors constant. This supports hypothesis *H*_3_.

**Table 10:** Regression results for transactional leadership.

**Variable**	**β**	**Standard error (SE)**	**t-value**	***p-*value**
Intercept	3.60	0.40	9.00	<0.001
Gender	−0.35	0.12	−2.92	0.005
Age	0.04	0.04	1.00	0.320
Years of experience	0.02	0.03	0.67	0.505
Academic rank	0.06	0.06	1.00	0.320

## 6 Discussion

The multiple regression models and other statistical analysis tools confirm that gender significantly influences leadership style preferences among faculty members at Taif University. Female faculty members exhibit a higher preference for transformational leadership, while male faculty members show a higher preference for transactional leadership. These findings highlight the need for gender-sensitive approaches in leadership development programs to enhance organizational effectiveness and inclusivity.

Prior studies have highlighted that gender can significantly influence leadership style preferences and perceptions (Eagly and Johnson, 1990; Northouse, 2018). Eagly and Carli (2003) found that women are more likely to exhibit transformational leadership behaviors, which emphasize vision, inspiration, and personal connections, while men tend to prefer transactional leadership, which focuses on structure, rules, and rewards.

We also found that female faculty members at Taif University have a more favorable view of transformational leadership styles. Research by Bass and Avolio (1994) supports the idea that transformational leadership is often more appealing to female due to its emphasis on collaboration, empowerment, and nurturing. This aligns with the hypothesis *H*_2_ that female faculty members prefer transformational leadership. Yukl (2013) also noted that transformational leadership fosters an inclusive environment, which is often valued more by female faculty members. In the context of Taif University, this preference could enhance academic collaboration and support among faculty members.

As a third objective of this study, we showed that male faculty members prefer transactional leadership styles over female faculty members. Existing literature, including studies by Burns (1978) and Bass (1985), indicated that transactional leadership is characterized by a focus on exchanges and performance-based rewards, which may align more closely with traditional male leadership roles. The hypothesis *H*_3_ that examined if male faculty members have a higher preference for transactional leadership is supported by these findings. Additionally, Ayman and Korabik (2010) indicated that cultural norms and expectations often drive men toward transactional leadership styles, emphasizing efficiency and clear authority structures.

Transformational leadership, preferred by female faculty members, may support sustainability by fostering an environment of continuous improvement and collaboration. On the other hand, transactional leadership, preferred by male faculty members, can contribute to sustainability through clear expectations and reward systems that drive performance.

Understanding these gender-based differences in leadership preferences is crucial for leadership development programs at Taif University. Training programs that incorporate both transformational and transactional leadership elements can cater to the diverse preferences of faculty members. According to Ely et al. (2011), leadership development initiatives that recognize gender differences and promote inclusive practices can lead to more effective and harmonious organizational environments.

The analysis of the given data demonstrated significant gender-based differences in leadership style perceptions among faculty members at Taif University. Female faculty members exhibit a stronger preference for transformational leadership, which is characterized by inspiration, motivation, and personal connection. In contrast, male faculty members show a greater inclination toward transactional leadership, which emphasizes structure, rewards, and performance monitoring. These findings should be contextualized within the broader cultural and societal norms of Saudi Arabia, where traditional gender roles may still prevail. Alomiri (2015) observed that in Saudi Arabia, leadership styles are often influenced by societal expectations, which can affect both male and female leadership behaviors and preferences. The preference for transformational leadership among female faculty members at Taif University might reflect a shift toward more inclusive and participative leadership practices in academia, whereas the preference for transactional leadership among male faculty members could indicate an adherence to conventional hierarchical structures. These different views of leadership practices at Taif University can enhance our understating toward sustainable education.

## 7 Conclusion

This study examined gender differences in perceptions of leadership styles and their impact on faculty motivation at Taif University. The quantitative data analysis confirmed that gender significantly influences leadership style preferences among faculty members at Taif University. The findings revealed that female faculty members preferred transformational leadership, while male faculty members favored transactional leadership. These results emphasize the need for gender-sensitive leadership development programs that cater to diverse preferences, promoting inclusivity and effectiveness in the academic environment.

The study has some limitations that open new research possibilities for the future. The sample size was one of the study's limitations. Although the sample size requirement was satisfied (Kothari, 2004), an increased number of samples would have made it possible to generalize the results with more precision. In addition, the present study is a case study that might be replicated considering other contexts and institutions.

The current investigation and the literature suggested that gender may have an impact on leadership. The analysis of this study is a key for leaders, not only in education sector, to consider differences in gender's views regrading leadership styles. In addition, this study suggested that decision makers should be aware that what appropriate for men might not appropriate for women in terms of leadership types. By adopting these findings, leadership practices can move toward sustainability, which is crucial for the long-term success of higher education institutions that ensures the continuous development of faculty, students, and resources, contributing to national growth and preparing students for future challenges. Researchers studying leadership can utilize the findings to provide a clearer picture of associated topics like healthcare leadership. Future research should further investigate these dynamics to enhance leadership effectiveness and faculty satisfaction in academic settings.

## Data Availability

The raw data supporting the conclusions of this article will be made available by the authors, without undue reservation.
